# Plasmodium falciparum Sexual Commitment Rate Variation among Clinical Isolates and Diverse Laboratory-Adapted Lines

**DOI:** 10.1128/spectrum.02234-22

**Published:** 2022-11-21

**Authors:** Lindsay B. Stewart, Aline Freville, Till S. Voss, David A. Baker, Gordon A. Awandare, David J. Conway

**Affiliations:** a Department of Infection Biology, Faculty of Infectious and Tropical Diseases, London School of Hygiene and Tropical Medicine, London, United Kingdom; b Department of Medical Parasitology and Infection Biology, Swiss Tropical and Public Health Institute, University of Basel, Basal, Switzerland; c West African Centre for Cell Biology of Infectious Pathogens (WACCBIP), Department of Biochemistry, Cell and Molecular Biology, University of Ghana, Accra, Ghana; Weill Cornell Medicine

**Keywords:** clinical isolates, differentiation, gametocyte, intraspecific variation, sexual development

## Abstract

Asexual blood-stage malaria parasites must produce sexual progeny to infect mosquitoes. It is important to understand the scope and causes of intraspecific variation in sexual commitment rates, particularly for the major human parasite P. falciparum. First, two alternative assay methods of measuring sexual commitment were compared to test a genetically modified P. falciparum line with elevated commitment rates inducible by overexpression of GDV1. The methods yielded correlated measurements with higher sensitivity and precision being achieved by one employing detection of the early gametocyte differentiation marker Pfs16. Thus, this was used to survey a diverse range of parasite lines and test each in multiple biological replicate assays in a serum-free medium supplemented with Albumax. There were differences among six recent clinical isolates from Ghana in their mean rates of sexual commitment per cycle, ranging from 3.3% to 12.2%. Among 13 diverse long-term laboratory-adapted lines, mean sexual commitment rates for most ranged from 4.7% to 13.4%, a few had lower rates with means from 0.3 to 1.6%, and one with a nonfunctional *ap2-g* gene always showed zero commitment. Among a subset of lines tested for the effects of exogenous choline to suppress commitment, there were significant differences. As expected, there was no effect in a line that had lost the *gdv1* gene and that had generally low commitment, whereas the others showed quantitatively variable but significant responses to choline, suggesting potential trait variation. The results indicated the value of performing multiple replicate assays for understanding the variation of this key reproductive trait that likely affects transmission.

**IMPORTANCE** Only sexual-stage malaria parasites are transmitted from human blood to mosquitoes. Thus, it is vital to understand variations in sexual commitment rates because these may be modifiable or susceptible to blocking. Two different methods of commitment rate measurement were first compared, demonstrating higher sensitivity and precision by the detection of an early differentiation marker, which was subsequently used to survey diverse lines. Clinical isolates from Ghana showed significant variation in mean per-cycle commitment rates and variation among biological replicates. Laboratory-adapted lines of diverse origins had a wider range with most being within the range observed for the clinical isolates, while a minority consistently had lower or zero rates. There was quantitative variation in the effects when adding choline to suppress commitment, indicating differing responsiveness of parasites to this environmental modification. Performing multiple assay replicates and comparisons of diverse isolates was important to understand this trait and its potential effects on transmission.

## INTRODUCTION

For malaria to be transmitted, asexual blood-stage parasites must undergo differentiation, which leads to the formation of male and female gametocytes that are required to infect mosquitoes. This process involves an initial parasite commitment phase when the switch to sexual development is induced, followed by a longer developmental phase to produce the mature gametocyte forms. From an evolutionary or ecological perspective, it is important to understand what determines the rate of commitment to sexual forms (1) and how this may vary within a species (2). It is particularly vital for understanding Plasmodium falciparum transmission as more effective control is needed to reduce the global malaria burden (3), which is particularly challenging as chronic asymptomatic infections are often highly infective to mosquitoes (4).

Research on malaria parasite sexual conversion has been long established (5), and progress has been made in elucidating key aspects of the mechanism. An ApiAP2-family transcription factor AP2-G is essential for sexual conversion and controlling the expression of genes critical to early gametocytogenesis (6–9). The *ap2-g* locus is clonally variably expressed, with histone 2 lysine 9 trimethylation (H3K9me3) marks of heterochromatin silencing indicating that its expression is epigenetically regulated (10). Conditional depletion of heterochromatin protein 1 (HP1) from the promoter was shown to increase *ap2-g* transcription and sexual conversion (11). Furthermore, in P. falciparum a gametocyte development protein GDV1, which was previously linked with sexual commitment (12), is involved in the removal of HP1 from the *ap2-g* locus such that overexpression of *gdv1* causes elevated sexual commitment rates (13).

The classic model is that sexual commitment occurs in the intraerythrocytic cycle before the cycle in which parasite sexual differentiation occurs, based on a finding that in static culture individual schizonts of the 3D7 parasite clone yield spatially clustered intraerythrocytic progeny that tends to differentiate similarly, either sexually or asexually (14). However, there is recent evidence that some sexual commitment may also occur in the same cycle as sexual differentiation if the *ap2-g* expression is induced very early after erythrocyte invasion during the ring stage (15), although it is not known how commonly this occurs in parasites under normal conditions of infection or laboratory culture. It is difficult to study P. falciparum gametocyte conversion rates precisely in natural infections because most stages of developing gametocytes do not occur in the peripheral circulation and are, therefore, not detected in blood samples. However, it may be possible to detect circulating ring-stage parasites that are sexually committed by developing assays for early markers such as AP2-G or GEXP02 (16–18). Correlations between P. falciparum gametocyte numbers and other measured variables in human hosts, such as age or hemoglobin levels, may not be causally related to various gametocyte conversion rates and are not well replicated across different studies (19). Experiments with other malaria parasite species in laboratory mice indicate a sexual commitment to be a plastic phenotype that can respond to the *in vivo* environment (20). Data relating to P. falciparum gametocyte conversion in experimentally induced human infections with the parasite clone 3D7 indicated that the conversion rate per cycle is usually less than 5%, and often less than 1% (18, 21). Older data on induced malaria infections, with P. falciparum strains that no longer exist, suggest similarly low rates of *in vivo* gametocyte conversion (22).

Early studies of P. falciparum in culture indicated that gametocyte production varied among parasite clones (23, 24) and varied with culture conditions, although precise estimates of conversion rates per cycle were not established. Subsequent studies estimated gametocyte conversion rates of several laboratory-adapted P. falciparum clones to be usually above 1% per cycle when grown at low parasitemia (6, 25), with higher rates if parasites were stressed at high parasitemia or by antimalarial drug treatment (14, 25). Parasites appear to respond to metabolic challenges during growth, although most cellular mechanisms remain to be identified (26). An increase in gametocytogenesis may be triggered by cellular stress involving redox perturbation (27, 28) by adding low doses of the antimalarial artemisinin at the early trophozoite growth stage (29) or by adding parasite-conditioned culture medium (30) or extracellular vesicles from such medium (31). There remains a need to understand which of these putative effects are artefactual or due to confounding in associations and which are reproducible. Notably, gametocyte conversion rates in cultured lines are suppressed by lysophosphatidylcholine (LysoPC) within the serum or by adding choline as a supplement in a serum-free medium (32, 33). In a study of parasite development during the initial *ex vivo* cycle cultured from clinical samples from Ghana, the proportions of parasites showing conversion to gametocytes correlated positively with the level of parasitemia and negatively with a concentration of LysoPC in plasma of patients, supporting a hypothesis that developmental responses to metabolic stress may occur during natural infections (34).

Despite these advances, the quantitative scope of sexual commitment rate variation in P. falciparum has not yet been clearly defined. Estimates of commitment rates have previously varied among different studies due to methodological differences or biological differences between nominally similar parasite lines, and there are few controlled comparisons of different isolates. Performing multiple independent replicate assays is important because some individual lines may have temporally variable phenotypes and precision is generally increased with repeated measurements. In this study, we first compared different methods of measuring sexual commitment rates and then employed the method with greater sensitivity and accuracy to conduct multiple biological replicate assays on recently adapted clinical isolates from Ghana as well as a broad selection of long-term laboratory-adapted lines representing most of those that are commonly cultured globally. Although each clinical isolate showed variability among repeated assays, there were also significant differences among isolates, indicating potentially adaptive variation in sexual commitment within this species. Separate assays of the suppressive effect on sexual commitment by adding choline to a serum-free culture medium identified significant quantitative variation among lines, which encourages research to test for relevance to disease transmission.

## RESULTS

### Comparison of sensitivity and precision of two different assays to measure parasite sexual commitment rates.

**FIG 1:**
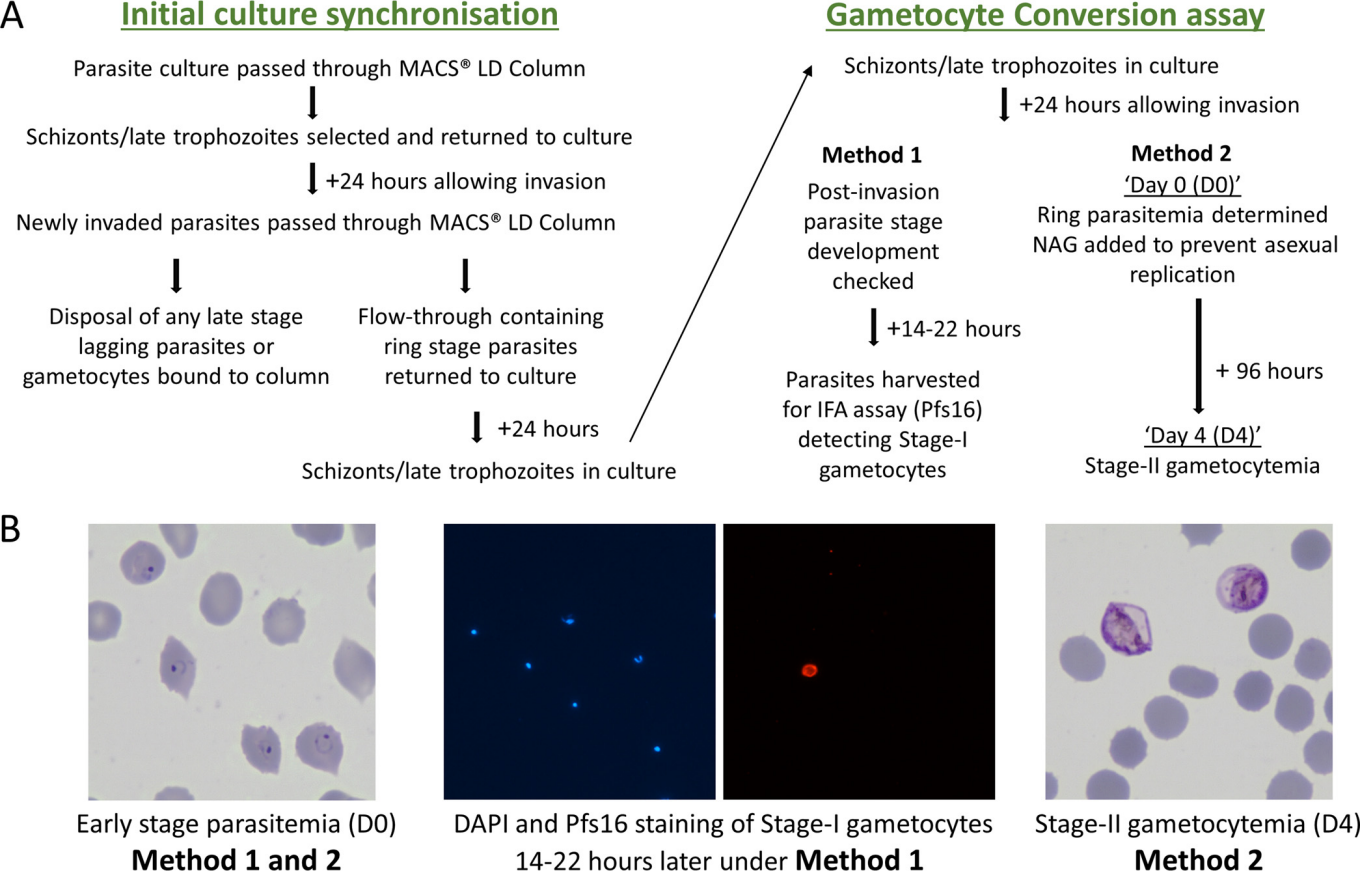
Comparison of two different methods to assay P. falciparum gametocyte conversion rates in culture (details in Materials and Methods). (A) Scheme outlining the timing of processes undertaken for the different methods, Assay Method 1 involving staining of Pfs16 as a marker of early gametocytes, and Assay Method 2 involving the counting of stage-II gametocytes after 4 days of development in cultures treated with *N*-acetyl-d-glucosamine (NAG) to prevent asexual parasite replication. (B) Representative microscopy images of parasites on Giemsa-stained slides used for counting ring stage parasitemia early in the cycle (day 0); counting of proportions of stage-I gametocytes 16 to 22 h later by staining of the early gametocyte differentiation marker Pfs16 (DAPI staining of parasite DNA in blue, and anti-Pfs16 monoclonal antibody staining in red) for Assay Method 1; and counting stage-II gametocytemia in the NAG-treated culture after 96 h (day 4) for Assay Method 2.

**FIG 2:**
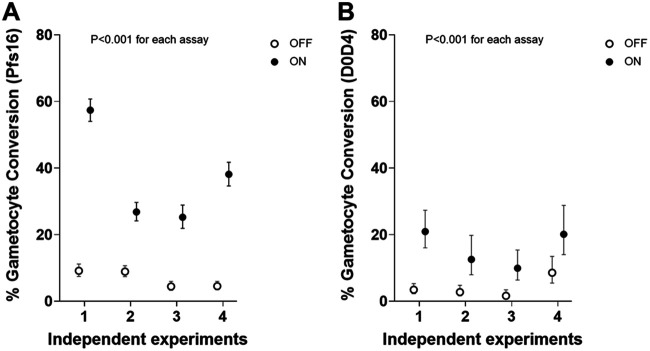
Comparison of the sensitivity and precision of two different methods for measuring P. falciparum sexual commitment in terms of gametocyte conversion rates. Multiple paired replicate assays were performed with the parasite clone 3D7/iGP_D9 (35), which allowed inducible overexpression of GDV1 fused to a destabilization domain (DD) stabilized in the presence of Shield-1 reagent to provide controlled elevation of sexual commitment (closed circles indicate GDV1–ON, open circles GDV1-OFF). Both methods showed the expected significant elevation in gametocyte conversion in the presence of Shield-1 reagent in all individual assays (*P* < 0.001 for each of the individual replicate assays). (A) The gametocyte conversion rate (with 95% CI) for each of seven independent experiments using Assay Method 1 involving staining of the gametocyte marker Pfs16, yielding a mean conversion rate of 38.3% under GDV1-ON and 6.3% under GDV1-OFF conditions (B) The gametocyte conversion rate (with 95% CI) for each of four experiments using Assay Method 2, which involved a comparison of day 4 (D4) gametocyte parasitemia with D0 ring-stage parasitemia (assays paired with the first four independent experiments shown for Assay Method 1), yielding a mean conversion rate of 15.9% under GDV1-ON and 4.1% under GDV1-OFF conditions. Exact parasite and erythrocyte count and statistical analyses for all assays are detailed in Table S1 in Supplemental File 1.

Two different assay methods were compared to quantify sexual commitment rates in multiple independent experiments, using a genetically engineered P. falciparum clone (3D7/iGP_D9) to overexpress GDV1 with a destabilizing domain (DD) in the presence of the stabilizing reagent Shield-1, which leads to elevated gametocyte conversion (35). Assay Method 1 involved differential counting of stage I gametocytes detected by anti-Pfs16 immunofluorescence staining as a proportion of all parasites developing beyond ring stages in the next cycle, while Assay Method 2 involved Giemsa-stained slide microscopy to compare the next cycle ring stage parasitemia (referred to as day 0 in this assay, D0) with the parasitemia of morphologically defined stage II gametocytes at day 4 (D4; Fig. 1; details in Materials and Methods). In each of the multiple biological replicate experiments, both methods clearly showed that the gametocyte conversion rate was significantly higher when GDV1 was overexpressed and stabilized with Shield-1 (GDV1-DD-ON) compared to when GDV1 overexpression was disabled by the absence of Shield-1 (GDV1-DD-OFF) (Fig. 2; Table S1 in Supplemental File 1).

In all biological replicate assays, Assay Method 1 showed a marked difference in gametocyte conversion rate between the GDV1-DD ON and OFF conditions, with a mean conversion rate of 36.9% under ON and 6.7% under OFF conditions (Fig. 2). In comparison, Assay Method 2 yielded an estimated mean conversion rate of 15.9% under ON and 4.1% under OFF conditions, with the rates for Assay Method 2 being lower than for Assay Method 1 in all replicates. The precision of the estimates for each of the biological replicates was higher for Assay Method 1, with relatively narrow confidence intervals for each experimental replicate because this method was based on direct counts of developmentally discrete parasites. In comparison, Assay Method 2 yielded wider confidence intervals because it was based on estimating the proportional difference of two temporally sequential counts of parasitemia, which involves more statistical sampling error (Table S1 in Supplemental File 1). This also depended on parasite development over a longer time in culture than required for Assay Method 1.

### Correlation between sexual commitment rate estimates from different assay methods.

**FIG 3:**
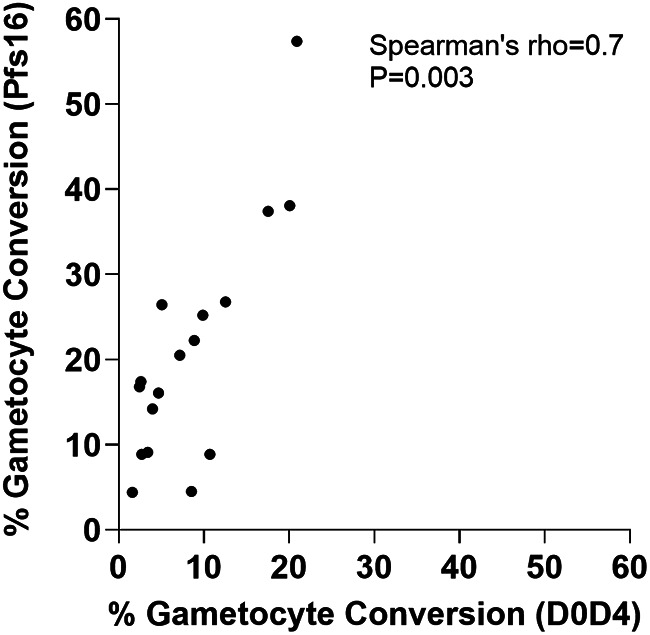
Significant correlation in the estimates of gametocyte conversion rate obtained by the two different assay methods being compared. Paired assays were performed using Assay Method 1 (involving Pfs16 staining to discriminate sexually developing from asexually developing parasites) and Assay Method 2 (involving counts of parasites 4 days apart to determine numbers of stage-II gametocytes compared to earlier ring stages) on multiple replicates of 3D7/iGP_D9 with a range of concentrations of Shield-1 reagent (N = 17 paired assays in total; details in Table S2 in Supplemental File 1). This significant correlation confirmed that either method gave valid measurements if used alone, whereas Assay Method 1 was more sensitive (as indicated here and in Fig. 2). Assay Method 1 was also more precise because it involved the calculation of a single numerical ratio rather than a ratio-of-ratios based on two different counts performed 4 days apart which is highly vulnerable to effects of small numbers in either count. For visual clarity, the individual assay 95% confidence intervals are not shown, but these and numerical data from all assays are in Table S2 in Supplemental File 1.

To test for overall correlation between the estimates from the two assay methods, analysis was performed on data from a broader range of paired assays. In addition to the eight measurements from paired assays performed in the first comparison (Fig. 2), nine additional measurements of the 3D7/iGP_D9 clone were performed with different concentrations of the Shield-1 reagent to yield a total of 17 paired measurements with both methods for analysis (Table S2 in Supplemental File 1). Overall, this showed a high and significant nonparametric rank correlation between the results from each assay method (Spearman’s rho = 0.70, *P* = 0.003), with Assay Method 1 generally giving higher conversion rate estimates (Fig. 3) with narrower 95% confidence intervals (Table S2 in Supplemental File 1). While this supported both assays as separately providing informative relative estimates of gametocyte conversion, Assay Method 1 was chosen for subsequent quantitative analyses of additional lines and culture conditions given its significantly higher sensitivity and tighter precision (Fig. 2 and 3, Table S1 and S2 in Supplemental File 1).

### Variation in sexual commitment rates of recent Ghanaian clinical isolates.

**FIG 4:**
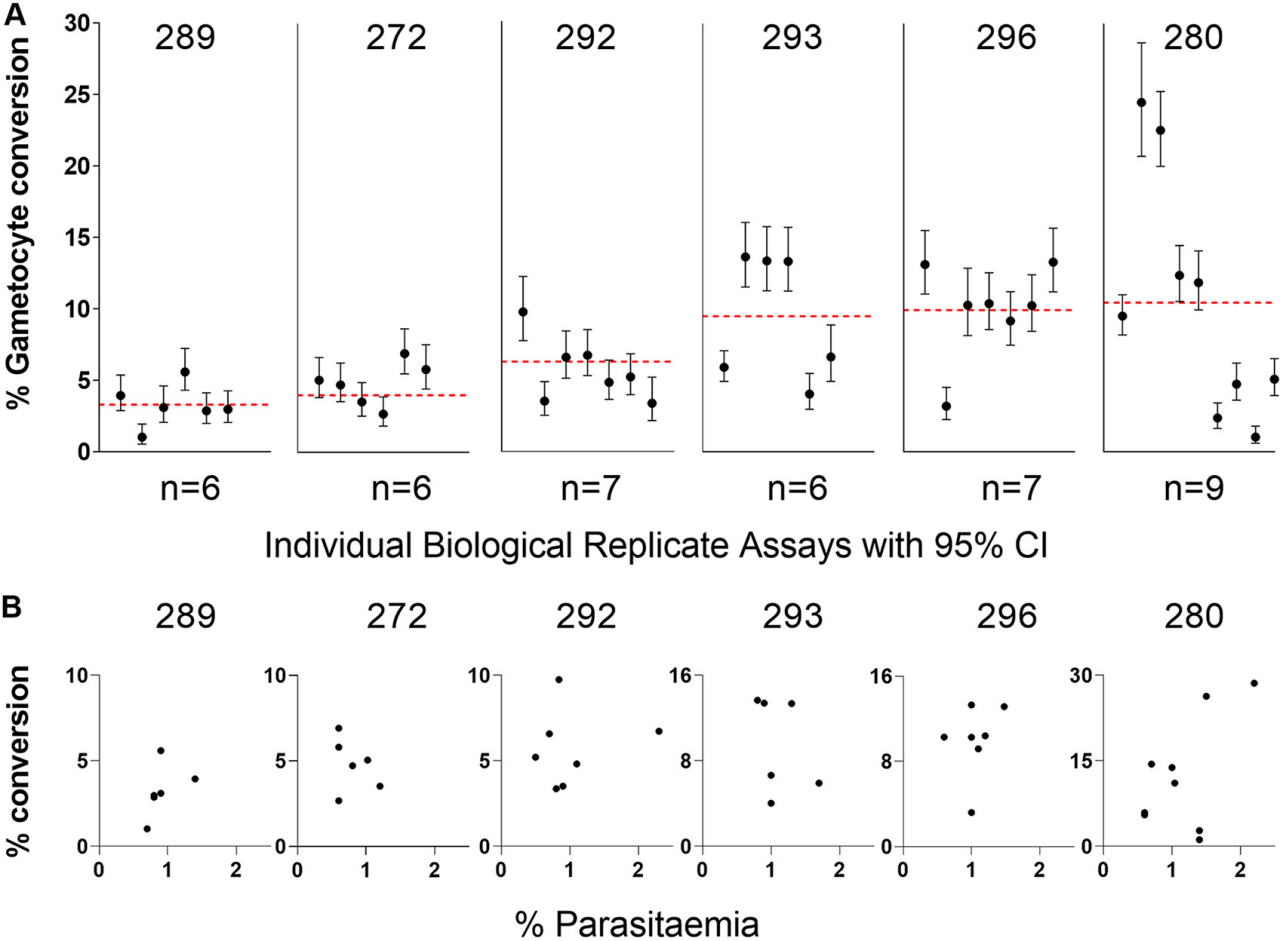
Gametocyte conversion rates of six recently culture-established Ghanaian clinical isolates. These were selected for study as they are unrelated single-genotype infections, chosen from a larger number of samples from a highly endemic population that contained many multiple-genotype infections (36). (A) For each isolate, a minimum of six biological replicate assays were performed at various times after different duration of culture (specified for each replicate in Table S3 in Supplemental File 1 and ranging between 48 to 125 days overall). The conversion rate estimate from each replicate is plotted with 95% confidence intervals, while the red dotted line represents the mean across replicate assays. There were significant differences in pairwise comparisons (Mann-Whitney tests, Table S4 in Supplemental File 1), with isolate 296 having a significantly higher conversion rate than three others (isolates 272, 289, and 292), and isolate 289 having a significantly lower rate than three others (292, 293, and 296) (Table S4 in Supplemental File 1). Details and all numerical data are given in Table S3 in Supplemental File 1. (B) For each isolate, the gametocyte conversion rate of individual replicates is plotted in relation to the culture parasitemia in the previous cycle (between 0.5 and 2.5% for all experimental replicates). The 95% confidence intervals for the individual conversion rate points are not shown because they are the same as in (A). There was no systematic trend toward positive or negative correlation, except a marginally significant positive correlation for isolate 289 (*P* = 0.02), which may indicate slight enhancement at higher parasitemia or a chance finding due to multiple comparisons (Table 1).

Recently culture-established clinical isolates from an area of high endemicity in Ghana were assayed to measure gametocyte conversion rates. Six isolates were selected from a larger panel for analysis because they represented genetically unmixed infections (36), and biological replicate assays corresponded to a single parasite genotype in each case. After culturing for several months, they did not have any known loss of function mutations detected in their genome sequences that would affect sexual commitment (36). These isolates showed mean gametocyte conversion rates that ranged from 3.3% to 12.2% (Fig. 4, Table S3 in Supplemental File 1), and there were significant differences in pairwise comparisons (Mann-Whitney tests on the results from a minimum of six biological replicate assays performed for each isolate; Table S4 in Supplemental File 1). For example, isolate 296 had a significantly higher conversion rate than three of the others (isolates 272, 289, and 292), and isolate 289 had a significantly lower rate than three of the others (292, 293, and 296) (Table S4 in Supplemental File 1).

There was also significant variation in gametocyte conversion rates among individual biological replicate assays of each isolate, with non-overlapping 95% confidence intervals of some replicates (Fig. 4A). Overall, the variation among biological replicate assays for each isolate was not correlated with the culture parasitemia at the beginning of the assay, which was always between 0.5% and 2.5%, except for a marginally significant positive correlation for isolate 289 (Fig. 4B; Table 1; Table S3 in Supplemental File 1). There was also no correlation with various levels of multiplication occurring within assays as assessed by fold change in parasitemia (Table 1; Table S3 in Supplemental File 1). Overall, there was no significant evidence of directional changes in gametocyte conversion rates over time, except for isolate 280 which had reduced rates after more than 71 days of culture (Table 1; Table S3 in Supplemental File 1). Variation among replicates did not correspond with the use of different erythrocyte batches for culture (Table S3 in Supplemental File 1).

**TABLE 1:** Tests for correlation of gametocyte conversion rates with measured variables in different experimental replicate assays for each of the cultured Ghanaian clinical isolates^*a*^

Isolate	No. of assays	Time of isolate in culture before assay		Parasitemia in the previous cycle		Parasitemia fold-change between cycles	
		rho	*P*	rho	*P*	rho	*P*
272	6	0.48	0.34	−0.33	0.52	0.60	0.21
280	9	*−0.75*	*0.02*	0.35	0.36	−0.27	0.49
289	6	−0.12	0.82	*0.88*	*0.02*	−0.24	0.65
292	7	−0.28	0.55	0.00	1.00	−0.54	0.22
293	6	−0.60	0.21	−0.59	0.22	0.41	0.43
296	7	0.19	0.69	0.30	0.52	−0.06	0.91
Mean		−0.18	0.44	0.10	0.44	−0.02	0.49

a Spearman’s ρ (rho) correlation coefficients and *P* values are shown for each isolate and mean values across isolates are shown at the bottom of the table. The gametocyte conversion rates are shown in Fig. 4. Correlations were nonsignificant, except for the two highlighted in italics (time in culture before assay for isolate 280, and parasitemia in the previous cycle for isolate 289). Across the different isolates, there was no directional trend for correlation with any variable. All variables and results for each replicate assay for each isolate are given in Table S3 in Supplemental File 1.

### Range of sexual commitment rates among diverse long-term cultured P. falciparum lines.

**FIG 5:**
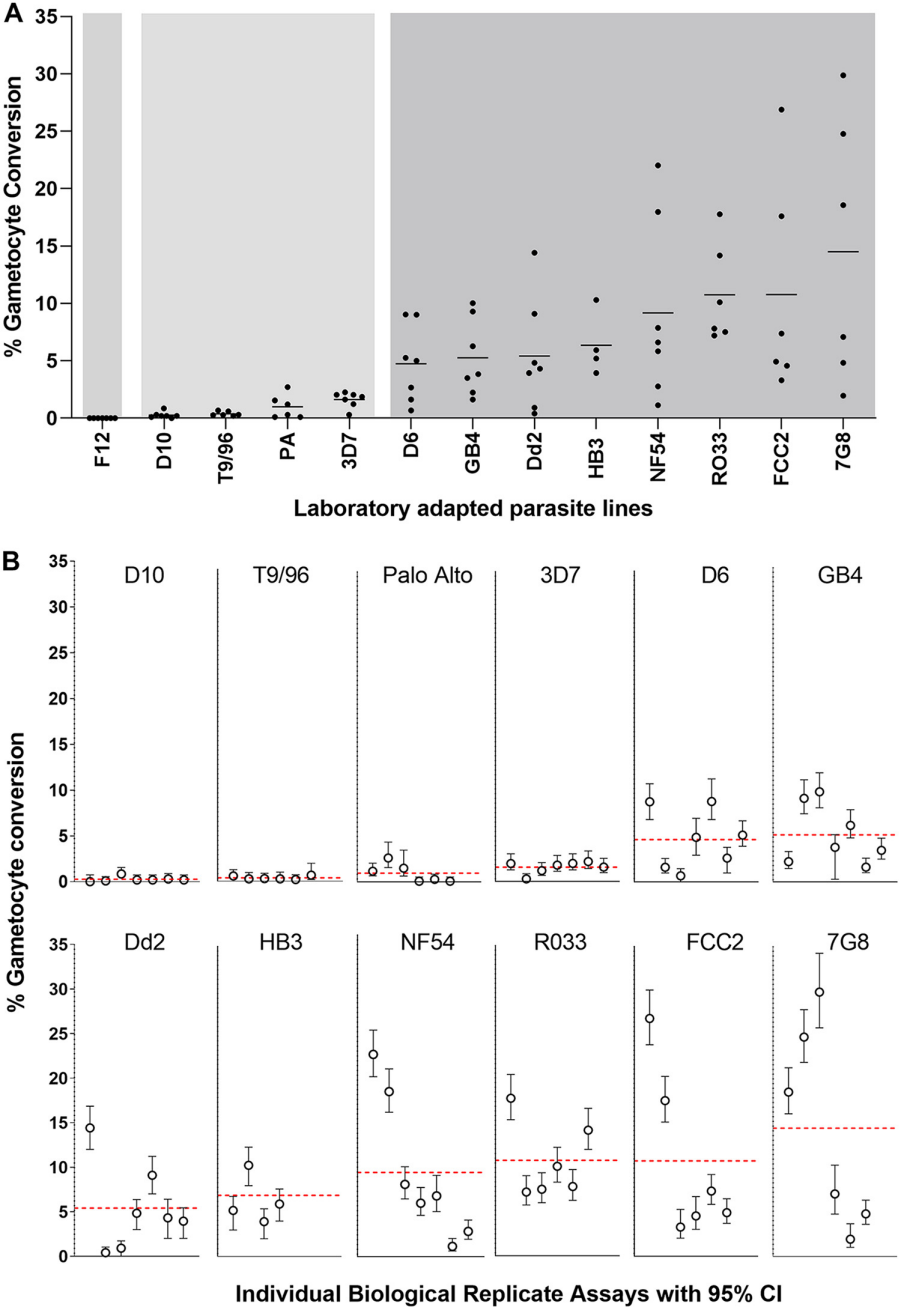
Gametocyte conversion rates of 13 long-term culture-adapted P. falciparum lines of diverse geographical origins. Each different parasite line was tested with multiple independent biological replicate assays, most individual lines having at least six replicates. All counts and calculated values for all replicates are given in full in Table S5 in Supplemental File 1. (A) For each parasite line, the conversion rates are shown for each of the biological replicates and the horizontal line shows the mean across all biological replicates, with a spectrum of variation among the different lines, means ranging from 0% to 14.5%. The background with different shades of gray show the conversion rates separating into three different groups, based on pairwise comparisons of all replicates between the parasite lines (Mann-Whitney tests, Table S6 in Supplemental File 1). One line (F12, with a non-functional *ap2-g* gene) shows no conversion, four lines (D10, T9/96, Palo Alto, and 3D7) show consistently low conversion (means between 0.3% and 1.6%), and eight lines (D6, GB4, Dd2, HB3, NF54, RO33, 7G8, and FCC2) shows higher levels of conversion (means between 4.7% and 14.5%). (B) Variation in the gametocyte conversion rates among independent replicate assays for each line is shown in more detail by, including 95% CI of the counts for each assay of each line, except F12 for which there was zero conversion in all assays. Identical methods were used in assaying these parasite lines as the clinical isolates shown in Fig. 4, with assays being conducted in parallel under the same conditions in the same laboratory. Erythrocytes from different erythrocyte donors used at various times throughout the series of experiments did not determine the variation between assay replicates (Table S5 in Supplemental File 1).

To compare with these recent clinical isolates, a panel of 13 long-term culture-adapted P. falciparum lines of diverse origins was tested, with multiple biological replicate assays (Fig. 5, Table S5 in Supplemental File 1). The clone F12 (containing a nonsense mutation in *ap2-g*) (6) showed zero conversion in all replicate assays (Fig. 5A; Table S5 in Supplemental File 1). Four of the other lines showed consistently low levels of gametocyte conversion, with means ranging between 0.3% and 1.6%, which was significantly lower than the conversion rates of the remaining lines (Mann-Whitney tests on pairwise comparisons, Table S6 in Supplemental File 1). The parasite lines with the lowest conversion rates (D10 and T9/96) were previously described as having multiple gene deletions in a subtelomeric region of chromosome 9 including the *gdv1* gene (37). The long-term cultured 3D7 line assayed here may have contained undetected mutants within the bulk culture because 3D7 cultured elsewhere was a source of parasite subclones with loss of function mutations (including the *ap2-g* nonsense mutation in parasite subclone F12 (6), deletion of *gdv1* in subclone 3D7.G_def_ (12) and truncation of *gdv1* in two other subclones (38)). The Palo Alto line was previously shown to have undergone subtelomeric gene deletions on different chromosomes (37, 39) but not to have lost the *gdv1* locus on chromosome 9 (37, 40), although the possibility of a loss-of-function mutation was not excluded.

For the remaining eight long-term culture-adapted P. falciparum lines, the gametocyte conversion rates for each showed means ranging from 4.7% (for D6) to 14.5% (for FCC2) (Fig. 5A). This range was similar to that for the recently culture-established Ghanaian clinical isolates described above, with no significant differences between these groups (Mann-Whitney test, *P* = 0.47). Similar to the clinical isolates, the individual long-term culture-adapted parasite lines also showed significant variation in gametocyte conversion rates among biological replicate assays (Fig. 5A), with non-overlapping 95% confidence intervals for some replicates (Fig. 5B). This showed that high and variable sexual commitment rates had been maintained in most of the diverse cultured P. falciparum lines, in contrast to a minority of lines that had markedly reduced rates or zero commitment.

Parasites were genotyped for a major dimorphism in *gdv1* (mutually exclusive large intergenic indels in the 3′-intergenic region in strong linkage disequilibrium with coding SNPs), previously been shown to have exceptional geographic divergence in allele frequencies (40). All six of the recent culture-established clinical isolates had the *3D7-like* allelic type of this dimorphism (the most common type within Ghana) (40) and three of the eight long-term culture-adapted P. falciparum lines with non-minimal gametocyte conversion rates also had the *3D7-like* allelic type (lines NF54, GB4 and D6). The other five of the long-term culture-adapted P. falciparum lines with non-minimal gametocyte conversion rates had the alternative *Dd2-like* allelic type (lines Dd2, HB3, RO33, FCC2, and 7G8). Among this limited set of clinical isolates and long-term established lines, the gametocyte conversion rates were not significantly associated with the *gdv1* genotype (mean conversion rate of 7.2% in parasites with the *3D7-like* allelic type and 9.7% in parasites with the *Dd2-like* allelic type; Mann-Whitney test, *P* = 0.15).

### Choline in culture medium suppressed sexual commitment with various effects among P. falciparum lines.

**FIG 6:**
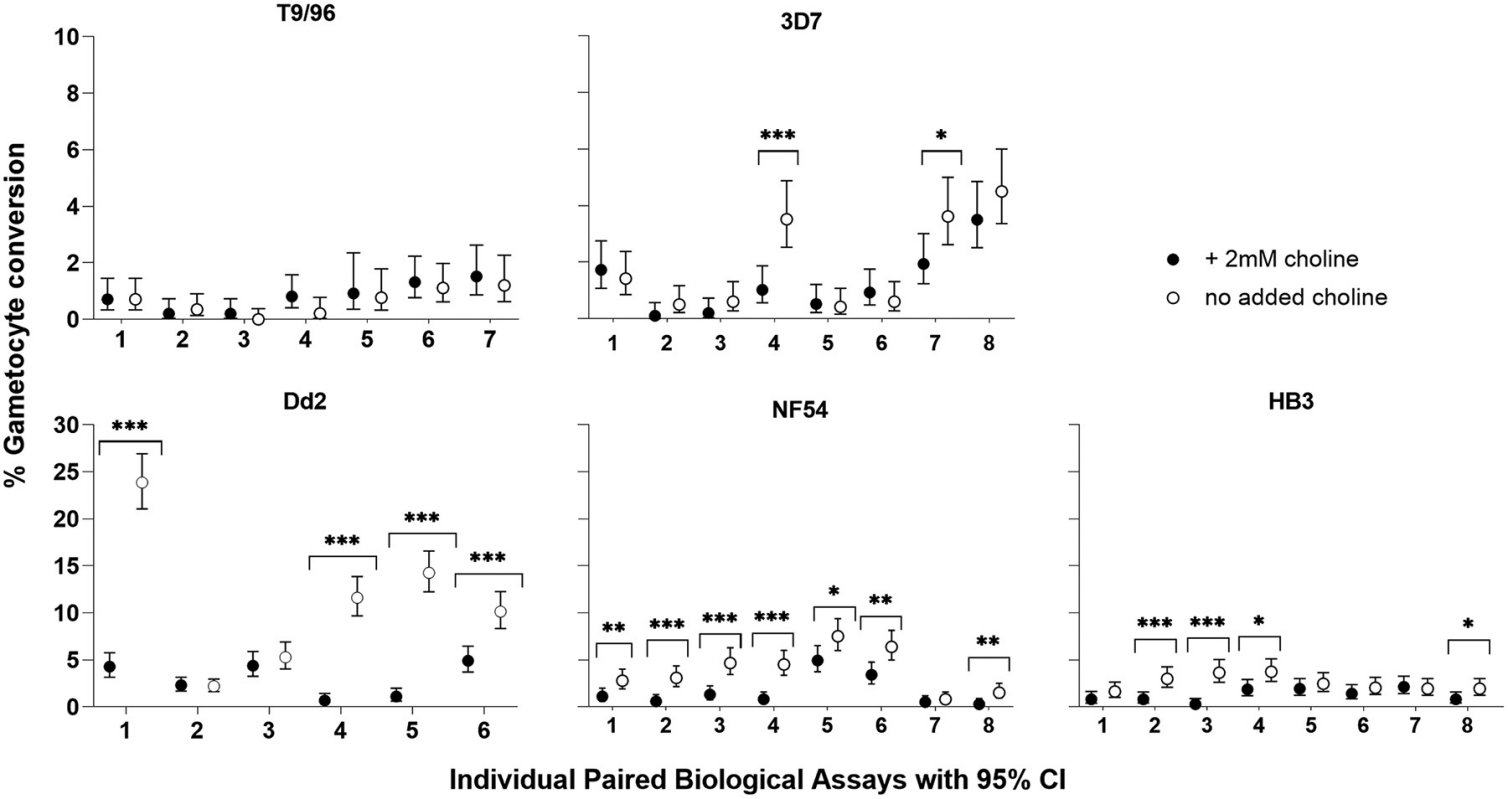
Variation in sensitivity of different P. falciparum lines to choline in medium to suppress sexual commitment. Closed circles represent biological replicates cultured in the presence of 2 mM choline, and open circles represent biological replicates grown in the absence of choline. T9/96 showed no response to choline in any of the seven biological replicate assays, whereas each of the other cultured lines showed responses that vary among biological replicates. Significant differences between paired measurements with and without choline in individual replicate assays for 3D7, Dd2, HB3 and NF54 are highlighted with asterisks (Fisher’s Exact Test; *, *P* < 0.05; **, *P* < 0.01; ***, *P* < 0.001). Across all replicates, estimated by Mantel-Haenszel rate ratios, the mean effect of choline was greatest for Dd2 (3.5-fold; 95% CI, 3.0- to 4.1-fold), intermediate for NF54 (2.4-fold; 95% CI, 2.0- to 3.0-fold) and HB3 (2.0-fold; 95% CI, 1.6- to 2.5-fold), and the least for 3D7 (1.5-fold; 95% CI, 1.2- to 1.9-fold). All individual counts and assay values for all biological replicates are in Table S7 in Supplemental File 1.

Because it had been previously shown that parasite sexual commitment responded to the presence or absence of choline in culture medium (13, 29, 32), we investigated five long-term culture-adapted lines (T9/96, 3D7, Dd2, NF54, and HB3) for quantitative effects of 2 mM choline compared to no choline added to a minimal culture medium (described in Materials and Methods). Between six and eight biological replicate assay comparisons were performed for each parasite line. Although there was variation among biological replicates, significant suppression of sexual commitment in the presence of choline occurred in most lines (Fig. 6; Table S7 in Supplemental File 1). No significant effect was seen in the P. falciparum clone T9/96, which had a large deletion of *gdv1* and distal adjacent genes on Chromosome 9 (37) and a low rate of sexual commitment either in the presence or absence of choline, consistent with the importance of *gdv1* (13). The responses of the other lines to choline varied with statistically significant suppression in sexual commitment in most of the replicate assays for NF54 and Dd2, in half of the assays for HB3, and a quarter of the assays for 3D7 (Fig. 6). Some individual assay replicates showed more than 10-fold suppression of sexual commitment rate in the presence of choline, but most showed more moderate rates. For each isolate, an assay was more likely to be significant when the gametocyte conversion rate in the control was high (Fig. 6; Table S7 in Supplemental File 1).

The variation in the suppressive effect of choline among replicate assays of each isolate was not correlated with culture parasitemia or estimated fold change in parasitemia over the cycle occurring within an assay (Table S7 in Supplemental File 1). The estimated fold changes in parasitemia also did not show an overall difference between treatments with choline or without choline (Table S7 in Supplemental File 1). The mean response of each parasite line to choline was estimated by the Mantel-Haenszel rate ratio (RR_M-H_), which analyzes data across replicates under a simple fixed-effects model for each line. Except for T9/96, which had minimal gametocyte conversion and showed no response to choline as noted above, the remaining four lines showed an overall significant reduction in gametocyte conversion rates with mean responses varying among the different lines (Table S7 in Supplemental File 1). The greatest mean response to choline was shown by line Dd2 (3.5-fold reduction; 95% CI, 3.0- to 4.1-fold), while 3D7 showed the lowest level of effect (1.5-fold reduction; 95% CI, 1.2- to 1.9-fold), and the other lines showed intermediate levels (for NF54, 2.4-fold reduction; 95% CI, 2.0- to 3.0-fold; for HB3, 2.0-fold reduction; 95% CI, 1.6- to 2.5-fold).

## DISCUSSION

Measuring the variable rates of sexual commitment in diverse isolates of P. falciparum is important. It is not feasible to always maintain parasites from different sources and with different phenotypes under identical conditions, so a priority of this study was to perform more biological replicate assays of each parasite line than usually performed and to analyze more lines than have been previously compared in continuous culture. A controlled comparison of two alternative assay methods here first demonstrated higher sensitivity and precision with an assay that measures proportions of sexually differentiating parasites by immunofluorescence staining of the Pfs16 marker expressed in early-stage I gametocytes (15). The other assay, which does not employ staining of an early gametocyte marker, requires parasites to successfully develop stage II gametocytes over 4 days in culture before they are counted (34).

Using the more sensitive assay method, the recently culture-established Ghanaian clinical isolates analyzed here had sexual commitment rates of between 3.3% and 12.2% per cycle, which were measured as means of the results from at least six biological replicate assays for each isolate. These rates were higher than those previously estimated for clinical isolates in the first cycle *ex vivo* in Ghana with an assay involving the counting of stage II gametocytes, which indicated most isolates had rates less than 1% and only a quarter of isolates had rates above 3% (34). The lower *ex vivo* rates might be partly due to lower assay sensitivity compared to the Pfs16 staining method, as well as the effects of plasma components, such as lysophosphatidylcholine, in some individuals inhibiting parasite sexual commitment (34). The clinical isolates in the present study were cultured consistently in a serum-free medium supplemented by Albumax-II for several months, and parasites preserved their intrinsic capacity to show robust rates of sexual commitment over this period.

Most of the diverse long-term laboratory-adapted lines had mean gametocyte conversion rates between 4.7% and 14.5% per cycle, a similar range to that seen for the cultured clinical isolates. The only line that always had zero sexual conversion was F12 with a previously described nonfunctional *ap2-g* gene due to a nonsense mutation (6). All other lines showed some detectable sexual conversion, although four lines had much lower rates (means of 0.3 to 1.6%) than the rest. This was likely due to loss-of-function mutations acquired in culture, including deletion of the *gdv1* gene in lines T9/96 and D10 (37), which showed the lowest conversion rates. Loss of *gdv1* in two other laboratory lines was reportedly associated with no detectable gametocytes (12, 38), and the low level of conversion in T9/96 and D10 may reflect a small amount of commitment to gametocyte development postinvasion due to stochastic activation of *ap2-g*, independent of the main pathway involving *gdv1* (15).

The replicate measurements of each P. falciparum line enabled the identification of significant variation among recently derived clinical isolates from the same local endemic population, as well as among long-term laboratory-adapted lines that have diverse geographical origins. Given that modeling of data from experimental infections has indicated much lower rates per cycle (21, 22), there may be a loss of parasites during the long period of development to circulating late-stage gametocytes *in vivo*, as well as suppression of gametocyte conversion during human infections compared to culture.

Variation in gametocyte conversion rates was seen among biological replicate assays of each parasite line, similarly for recently established clinical isolates as for most of the long-term laboratory-adapted lines. Measured differences among replicates may reflect intrinsic parasite variation, stochasticity, or determinants needing to be identified that are affected by variation in assay conditions. Overall, there were no systematic correlations with measured variables among replicates in this study, including the length of time clinical isolates had been in culture, the level of culture parasitemia at the initiation of the assay, or the fold change in parasitemia over the cycle during the assay. Some studies on several different culture-adapted P. falciparum clones have shown less variation between experimental replicates (6, 13, 29, 32), which may reflect the employment of a tighter parasite stage synchronization in those studies, whereas a broad window of synchronization for setting up assays in the present study was intended to reflect the age range of parasites that may be sampled in peripheral blood. By the end of assays here, although most parasites would be mature enough for assessment of sexual commitment by Pfs16 staining, some replicates would have most parasites at least 36 h postinvasion when all stage I gametocytes would be Pfs16-positive (15, 17, 41), while other replicates may have some parasites only 24 h postinvasion when a majority but not all stage I gametocytes may already be Pfs16-positive (15). Exact standardization of parasite ages in the culture of diverse isolates is not feasible because isolates intrinsically vary in developmental cycle times (42, 43), but tighter synchronization of parasite cultures is likely to reduce variation among replicates for any individual isolate. Assay sensitivity might also be increased further if detection assays are developed to enable staining of sexually committed parasites before they express Pfs16, which may become possible as particular gene transcripts are expressed in committed ring stages (18), and at least one protein (GEXP02) is expressed earlier than Pfs16 (17).

Although most of the diverse laboratory-adapted parasite lines tested in this study retain an intact mechanism for sexual commitment, there were differences in responses to the suppressive effects of choline. The presence or absence of choline had no effect on the T9/96 clone that has a culture-acquired deletion of *gdv1*, consistent with the requirement of this locus for regulating levels of sexual commitment (12, 13). In contrast, choline had a significant but variable effect on sexual commitment in each of the other parasite lines tested. Although a few individual replicate assays showed greater than 10-fold effects, the mean suppressive effects of choline in each parasite line ranged from 1.5-fold (for 3D7) to 3.5-fold (for Dd2). These effects are more moderate than described in previous studies of a few parasite clones that had been maintained in a medium containing choline or serum before being switched to a choline-free medium (13, 17, 32). Gametocyte conversion rates of >30% have occasionally been reported previously (29), which were not seen here except after overexpression of GDV1 in an engineered line. Because all parasites in this study were maintained in a standard culture medium with Albumax without choline or serum supplementation, culture parasites with exceptionally high conversion rates might have been selected without suppression of gametocyte conversion over time (32). It also cannot be excluded that unknown mutations affecting the regulation of sexual commitment may have been acquired during the recent culture of some of the parasite lines studied here because, although parasite genomes were previously sequenced and analyzed (36, 44), they had not been resequenced from sampling again at the time of each of the assays.

The results support the utility of choline in a medium to suppress parasite sexual commitment under controlled environmental conditions (10, 20, 21) and show significant variation in the responsiveness of different parasite lines. It will be important to understand if there is intraspecific variation in the natural regulation of GDV1 expression, particularly the control of gene-silencing antisense transcripts (13), and the potential influence of naturally occurring cis-variation at the subtelomeric locus on chromosome 9 (34, 40, 45) distinct from the deletions disrupting function in some cultured lines (12, 37, 38, 46). Although the major allelic dimorphism at the *gdv1* locus was not significantly associated with variability in gametocyte conversion rates in the present study, the number of isolates investigated was limited, and the panel was not chosen to test for such an association. It has also recently been reported that sexual commitment is affected by competition between histone methyltransferases (involved in *ap2-g* gene silencing) and phosphoethanolamine methyltransferase (involved in *de novo* synthesis of phosphatidylcholine when choline availability is low) for the methyl donor S-adenosylmethionine, which could have implications for future studies on variation in rates of sexual commitment (33). Analyses of parasites from natural populations benefit from performing multiple assay replicates, so this needs consideration alongside the need to increase the number of isolates examined in future candidate gene studies and genome-scale scanning for allelic associations (47).

## MATERIALS AND METHODS

### Ethics approval.

Approval for the sampling of clinical isolates for culture experimental analysis was granted by the Ethics committees of the Ghana Health Service, the Noguchi Memorial Institute for Medical Research at the University of Ghana, the Navrongo Health Research Centre, and the London School of Hygiene and Tropical Medicine, as previously described for the collection (36) from which specific isolates were selected in this analysis.

### Plasmodium falciparum clinical isolates and laboratory-adapted clones.

Six P. falciparum clinical isolates from Ghana with single-genome unmixed infections were selected from a larger panel of infections sampled from children with clinical malaria in Navrongo (in the Upper East Region of the country), in which parasite asexual multiplication rate variation was previously studied (36). These isolates were cultured in commercially prepared serum-free medium supplemented with lipid-rich bovine serum albumin (Gibco RPMI with 0.5% AlbuMAX II lot number 2177881). This consistent medium preparation had minimal choline (less than 0.02 mM choline chloride), which would not suppress parasite sexual commitment. This was unlike high concentrations of choline (2 mM as used for some assays described in a separate section below) or serum, which had variable components between batches. These clinical isolates were not cultured with added serum at any time as they were cultured with 0.5% AlbuMAX II since isolation (36). All isolates were thawed from pre-existing frozen stocks and cultured for at least 1 week after thawing before any assay, and each was assayed after multiple different time points in culture since isolation from patients (ranging between 48 to 125 days).

Clinical isolates with mixed genome infections were purposefully not selected for inclusion in the present study because their sexual commitment rates would be complex to interpret due to changes in mixed genomic composition over time as previously described (36, 48). The six single-genome infection isolates studied here had a range of asexual multiplication rates covering most of the range seen in the total panel of isolates from which they were selected (36). The individual isolates were genetically unrelated to each other, as shown by pairwise comparisons based on a genome-wide analysis of 40,000 single nucleotide polymorphisms (36).

The genetically modified P. falciparum cloned line 3D7/iGP_D9 was used to compare the sensitivity and precision of two different gametocyte conversion rate assays (described in the following subsection). The artificially elevated sexual commitment was selectively inducible in this line by overexpression of GDV1 fused to a destabilization domain (DD), which maintained activity in the presence of Shield-1 reagent (35).

A diverse panel of 13 different P. falciparum long-term laboratory-adapted lines that have not undergone targeted genetic modification were studied. Each of these was either previously derived by cloning or was a line that has a monoclonal single-genome sequence, and a putative origin based on the geographical location of the original isolation: NF54 (a strain of ‘airport malaria’ from The Netherlands with genome sequence similarity to African isolates), 3D7 (the P. falciparum reference genome strain originally cloned from NF54 and maintained in culture for many years) (49), F12 (derived by cloning from cultured 3D7 and having a nonsense mutant *ap2-g* gene) (6), D10 (Papua New Guinea), D6 (Sierra Leone), Dd2 (Southeast Asia), FCC2 (China), HB3 (Honduras), R033 (Ghana), Palo Alto (Uganda), 7G8 (Brazil), GB4 (Ghana), and T9/96 (Thailand). These parasite lines were cultured in commercially prepared serum-free medium with 0.5% AlbuMAX II, using the same batch as for the clinical isolates, so that no biological variation should be due to medium components. All lines were thawed from pre-existing frozen stocks with distinct identities verified by previous targeted genotyping and sequencing (40, 50) without any recloning before this study, and each line was cultured for at least 1 week after thawing before any assay.

### Assays to quantify parasite sexual commitment rates.

Multiple biological replicate preparations of recently culture-established P. falciparum clinical isolates and long-term laboratory-adapted lines were cultured in human erythrocytes at 3% hematocrit in RPMI 1640 medium supplemented with AlbuMAX II and incubated at 37°C in atmospheric air with 5% CO_2_. Before assay, each asynchronous parasite culture was synchronized within a 24-h window of intraerythrocytic asexual stage development, which was intended to correspond approximately to the parasite age range that would be typically sampled in the peripheral blood of patients, using the following two-step technique. Parasites containing hemozoin, predominantly schizont, and late trophozoite stages, were first positively selected on MACS LD magnetic columns (51). After culturing for 24 h, a second MACS purification step was performed from which the flow through erythrocytes (containing predominantly ring stage parasites) were collected and put back into culture, which then contained exclusively ring stage parasites because any gametocytes or residual mature asexual parasites were removed on the column. This broad synchronization scheme is illustrated in Fig. 1. For the 3D7/iGP parasite line, the effect of GDV1-DD overexpression was tested by adding Shield-1 reagent to this preparation of ring stages (1 μM compared to none, with intermediate concentration treatments of 0.05, 0.15, and 0.5 μM Shield-1 also being tested in some assays), with Shield-1 being retained in the culture throughout the cycle.

The following two methods were used for measuring the gametocyte conversion rate.

### Assay Method 1: counting the proportion of stage I gametocytes compared to all parasites developing in the first cycle postinvasion using anti-Pfs16 staining.

This method involved distinguishing very early gametocytes from asexual parasites by detection of the gametocyte marker Pfs16 protein (15, 41, 52), as illustrated in Fig. 1. After two rounds of MACS column purification to obtain ring stages followed by a further 24 h of growth, when a culture with broad synchronicity containing schizonts was obtained, parasites were allowed to invade erythrocytes. They were cultured for a period of between 38 and 46 h (parasitaemia being checked after 24 hours, and cultures then continued for another 14–22 hours), the exact time of harvesting of each culture decided based on microscopic examination of Giemsa stained slides, so that most parasites would be either trophozoites or stage I gametocytes. The harvested parasites were examined by fluorescence microscopy for Pfs16 staining of early gametocytes and DAPI staining of all parasites. Stage I gametocytes were counted as those staining positive for Pfs16 and showing typical round morphology similar to trophozoites. Any Pfs16-positive parasites with differentiated morphology of stage II gametocytes that might have been residual from induction before the previous cycle were not counted. The assay allowed most sexually developing parasites to be counted in comparison to the total of postinvasion parasites because most of the early stage I gametocytes express Pfs16 after 24 h postinvasion (15). Parasite cultures harvested for this purpose were washed in phosphate-buffered saline (PBS) with 3% bovine serum albumin (BSA), resuspended to 2% hematocrit, spotted onto multiwell slides (Hendley, Essex, UK), air-dried, and stored at −80°C. Before immunofluorescence staining, slides were fixed with 4% paraformaldehyde in PBS for 30 min and permeabilized with 0.1% Triton X-100 in PBS for 10 min. The slides were incubated for 30 min at room temperature with a monoclonal α-Pfs16 murine antibody 93A3A2 (52) and diluted 1:2000 in PBS with 3% BSA. Alexa Fluor 594-conjugated anti-mouse IgG (Invitrogen, A11032) was used as a secondary antibody, diluted 1:1000 in PBS with 3% BSA, and incubated for 30 min at room temperature. Slides were mounted with coverslips using DAPI-containing Prolong^TM^ Diamond antifade mountant (ThermoFisher Scientific), and parasite counting was performed using a Zeiss CCD fluorescence microscope, with identical settings used for each experiment. An average of approximately 1000 parasites were counted to determine proportions expressing Pfs16 in each measurement, with at least 300 parasites being counted even in cases when parasite density on a slide was low.

### Assay Method 2: counting the developing gametocytemia compared to all parasites at the ring stage culture.

This method estimated the proportion of parasites that produced gametocytes developing to morphological stage II by comparing with the ring parasitemia counted 4 days previously at the beginning of the parasite developmental cycle. The technique was based on a protocol that used Giemsa staining and did not require immunofluorescence staining of an early gametocyte marker (34). To initiate this assay, after two MACS column purifications and when a broadly synchronous culture containing a majority of schizonts was obtained, parasites were allowed to reinvade fresh erythrocytes and cultured until ring-stage development was observed. Then, ring-stage parasitemia was assessed by visual examination of thin blood smears of each culture to determine the proportion of erythrocytes containing parasites (referred to as D0 parasitemia), ensuring that at least 1,000 erythrocytes were counted. The cultures were then treated with 50 mM *N*-acetyl-d-glucosamine (GlcNAc, abbreviated as NAG) to prevent the further development of asexual parasites (53). After 4 days, the stage II gametocytemia was assessed by counting Giemsa-stained thin blood smears (referred to as D4 gametocytemia), ensuring that at least 10,000 erythrocytes were counted. The gametocyte conversion rate was calculated by dividing D4 stage II gametocytemia (as a proportion of erythrocytes infected) by D0 ring parasitemia (as a proportion of erythrocytes infected), yielding a ‘ratio-of-ratio’ proportion with 95% confidence intervals.

For both methods, all assays were set up at 3% hematocrit in a culture volume of 5 mL (static in 6-well culture plates) with parasitemia levels at the start of each replicate assay being between 0.5 and 2.5% erythrocytes infected to allow sufficient numbers of parasites for high precision counting while minimizing stress on parasites that may be induced if cultures have higher parasitemia.

### Quantification of gametocyte conversion rate using culture medium with added choline.

Parasites were prepared for assays in the same manner as described in the previous section, except that they were split after the second MACS column purification (at the ring stage in the cycle before gametocyte counting) into separate wells with a minimal culture medium (RPMI 1640 with 25 mM HEPES, 100 μM Hypoxanthine, 1 mM l-glutamine, 0.39% fatty acid-free BSA, 30 mM Palmitic acid, 30 mM Oleic acid) either supplemented with 2 mM choline or lacking choline, based on a previously described protocol (32). Parasites were allowed to grow until the schizont stage and to invade fresh erythrocytes, without changing the culture medium. After 38 to 46 h, parasites were collected and fixed for analysis of gametocyte conversion by differential counting using Pfs16 staining by immunofluorescence microscopy, as well as DAPI staining, as described above for Assay Method 1, with an average of approximately 1000 parasites being counted for each measurement. Between six and eight replicate assays were performed for each parasite line, the effect within each replicate being calculated as the rate ratio (with 95% confidence intervals) of the gametocyte conversion rate in a medium containing 2 mM choline compared with the conversion rate in a choline-free medium.

### Genotyping of allelic dimorphism at the gdv1 locus.

Parasites were genotyped by analysis of extracted DNA, to determine the major dimorphic allelic types in the *gdv1* 3′-intergenic sequence, which have an exceptionally high level of geographical frequency divergence (40). These alleles comprised mutually exclusive large intergenic indels, either *3D7-like* or *Dd2-like*, and were in strong linkage disequilibrium with SNPs in the coding sequence of *gdv1*, which also have exceptional geographical frequency divergence (40, 54). Two different pairs of primers were used, one for each of the indels, in separate PCRs using the amplification conditions and electrophoretic scoring method as described previously (40).

### Statistical analyses.

Spearman’s ρ (rho) coefficient was used to evaluate the strength and significance of the rank correlation between results from the two different gametocyte conversion rate assays performed in parallel. Statistical comparisons of gametocyte conversion rate variation between different P. falciparum lines were performed using the Mann-Whitney test on the multiple replicate assay measurements of each line. All *P* values given corresponded to two-tailed tests of significance. Each different parasite line was assayed with a mean of more than six biological replicate cultures with most individual lines having at least six and some up to nine biological replicates performed. Each biological replicate data point had 95% confidence intervals calculated for the proportions of parasites showing sexual commitment based on the raw numerical counts performed, enabling accurate estimation of within-assay sampling variation, and all count data were provided for verification and to enable future meta-analyses. For each parasite line, the mean effect of modified culture medium conditions were estimated by calculating the Mantel-Haenszel rate ratio (RR_M-H_) across biological replicate assays, based on a simple fixed-effects model. Statistical analyses were performed using Prism version 9 and Epi-Info version 7 software.
