# Supplementary material for: Plasmodium falciparum Sexual Commitment Rate Variation among Clinical Isolates and Diverse Laboratory-Adapted Lines
Source: Microbiol Spectr. 2022 Nov 21;10(6):e02234-22. doi: 10.1128/spectrum.02234-22 (PMC9769538; doi:10.1128/spectrum.02234-22)
Supplement: Supplemental file 1 — Tables S1 to S7. Download spectrum.02234-22-s0001.pdf, PDF file, 0.5 MB [file spectrum.02234-22-s0001.pdf]

Supplementary Information

Supplementary Table S1. Gametocyte conversion rate (GCR) estimates (with 95% confidence intervals) and all numerical count data for two different methods with multiple experimental replicates applied to *P. falciparum* line 3D7/iGP under elevated GDV1 expression with Shield-1 reagent (+SHLD) and control (-SHLD) conditions.

		Method 1			Method 2		
Replicate	Condition	Pfs16 +ve	DAPI +ve	Pfs16 GCR (95%CI)	D0 % Parasit.	D4 % Parasit.	D0D4 GCR (95% CI)
1	-SHLD	84	921	9.12 (7.4 - 11.2)	10.1 (101/999)	0.3 (26/8262)	3.4 (2.2 -5.2)
	+SHLD	474	826	57.4 (54.0 - 60.7)	9.8 (98/999)	1.9 (117/6124)	20.9 (16.0 - 27.3)
2	-SHLD	104	1171	8.9 (7.4 - 10.7)	7.2 (67/932)	0.2 (15/8210)	2.7 (1.6 - 4.7)
	+SHLD	262	978	26.8 (24.1 - 29.7)	3.0 (29/970)	0.4 (49/13415)	12.5 (7.9 - 19.8)
3	-SHLD	41	926	4.4 (3.3 - 6.0)	5.6 (53/946)	0.1 (8/9305)	1.6 (2.8 - 3.4)
	+SHLD	150	595	25.2 (21.9 - 28.9)	4.4 (42/957)	0.4 (36/8625)	9.9 (6.3 - 15.4)
4	-SHLD	47	1043	4.5 (3.4 - 6.0)	5.0 (48/951)	0.4 (30/7278)	8.5 (5.4 - 13.5)
	+SHLD	267	701	38.1 (34.6 - 41.7)	4.4 (42/952)	1.0 (77/8070)	20.1 (14.0 - 28.8)

Supplementary Table S2. Estimates of gametocyte conversion rate (GCR) for 17 assays of the *P. falciparum* inducible 3D7/iGP_D9 line in which two different methods were performed in parallel for comparison (as detailed in Materials and Methods). Varying concentrations of Shield-1 (0.055 – 1.0 µM, and none) were used for the assays. Data are plotted and analysed in Figure 2, showing an overall correlation between the methods, with Method 1 being generally more sensitive (and more precise with narrower confidence intervals as shown here).

**Independent assays of *P. falciparum* line**	**Method 1 Pfs16 GCR (95%CI)**	**Method 2 D0/D4 GCR (95%CI)**
iGP - SHLD	9.1 (7.4 - 11.2)	3.4 (2.2 - 5.3)
iGP +SHLD (1.0)	57.4 (54.0 - 60.7)	20.9 (16.0 - 27.31)
iGP - SHLD	8.9 (7.4 - 10.7)	2.7 (1.6 - 4.8)
iGP + SHLD (0.055)	14.2 (12.3 - 16.4)	4.0 (2.2 - 7.2)
iGP + SHLD (0.15)	16.8 (14.8 - 19.1)	2.4 (1.2 (4.9)
iGP + SHLD (0.5)	17.4 (15.3 - 19.7)	2.6 (1.3 - 5.2)
iGP +SHLD (1.0)	26.8 (24.1 - 30.0)	12.5 (8.0 - 19.8)
iGP - SHLD	4.4 (3.4 - 7.0)	1.6 (0.8 - 3.4)
iGP + SHLD (0.055)	20.5 (17.9 - 23.4)	7.2 (4.6 - 11.3)
iGP + SHLD (0.15)	16.1 (13.8 - 18.7)	4.7 (3.2 - 6.9)
iGP + SHLD (0.5)	26.5 (23.5 - 29.6)	5.1 (3.2 - 8.2)
iGP +SHLD (1.0)	25.2 (21.9 - 28.9)	9.9 (6.4 - 15.4)
iGP - SHLD	4.5 (3.4 - 6.9)	8.5 (5.4 - 13.5)
iGP + SHLD (0.055)	8.9 (7.2 - 11.2)	10.7 (7.3 - 15.7)
iGP + SHLD (0.15)	22.3 (19.2 - 25.8)	8.9 (6.1 - 12.8)
iGP + SHLD (0.5)	37.4 (34.0 - 41.0)	17.5 (12.9 - 23.8)
iGP +SHLD (1.0)	38.1 (34.6 - 41.7)	20.1 (14.0 - 28.8)

Supplementary Table S3. Multiple biological replicate assays of gametocyte conversion rates (GCR) for each of 6 cultured *P. falciparum* clinical isolates from Navrongo, Ghana.

Isolate	Replicate	RBC Batch	Days in culture	% Parasitaemia Cycle before	% Parasitaemia post-invasion	Parasitaemia fold-change	Pfs16 +ve	DAPI +ve	GCR (95%CI)
289	1	12	92	1.4	4.4	3.1	38	961	4.0 (2.9 - 5.4)
	2	12	99	0.7	2.8	4.0	9	870	1.0 (0.6 - 2.0)
	3	12	99	0.9	2.9	3.2	23	740	3.1 (2.1 - 4.6)
	4	13	106	0.9	1.8	2.0	53	946	5.6 (4.3 - 7.3)
	5	13	106	0.8	2.1	2.6	28	971	2.9 (2.0 - 4.1)
	6	15	125	0.8	2.4	3.0	28	939	3.0 (2.1 - 4.3)
	Mean (SD)								*3.3 (SD = 1.5)*
272	1	12	83	1.0	1.0	1.0	48	951	5.0 (3.8 - 6.6)
	2	12	83	0.8	1.9	2.4	45	954	4.7 (3.5 - 6.3)
	3	13	91	1.2	1.5	1.2	34	965	3.5 (2.5 - 4.9)
	4	13	91	0.6	1.2	2.0	26	973	2.7 (1.8 - 3.9)
	5	15	110	0.6	6.3	10.5	69	999	6.9 (5.5 - 8.7)
	6	15	110	0.6	5.5	9.2	51	880	5.8 (4.4 - 7.5)
	Mean (SD)								*4.8 (SD = 1.5)*
292	1	1	67	0.8	0.8	1	66	677	9.7 (7.7 - 12.2)
	2	12	62	0.9	1.3	1.4	34	965	3.5 (2.5 - 4.9)
	3	12	62	0.7	1.4	2.0	58	882	6.6 (5.1 - 8.4)
	4	13	69	2.3	3.8	1.6	63	936	6.7 (5.3 - 8.5)
	5	13	69	1.1	2.1	1.9	46	953	4.8 (3.6 - 6.4)
	6	15	88	0.5	1.3	2.6	49	939	5.2 (4.0 - 6.8)
	7	15	88	0.8	4.0	5	19	564	3.4 (2.2 - 5.2)
	Mean (SD)								*5.7 (SD = 2.2)*
293	1	1	67	1.7	1.7	1	109	1841	5.9 (4.9 - 7.1)
	2	12	55	0.8	3.0	3.7	120	878	13.7 (11.6 - 16.1)
	3	13	69	0.9	2.1	2.3	118	881	13.4 (11.3 - 15.8)
	4	13	69	1.3	2.3	1.8	118	883	13.4 (11.3 - 15.8)
	5	13	76	1.0	2.7	2.7	39	962	4.1 (3.0 - 5.5)
	6	13	76	1.0	1.7	1.7	41	617	6.6 (4.9 - 8.9)
	Mean (SD)								*9.5 (SD = 4.4)*
296	1	12	48	1.5	2.8	1.9	116	883	13.1 (11.1 - 15.5)
	2	12	55	1.0	2.5	2.5	31	968	3.2 (2.3 - 4.5)
	3	12	55	0.6	1.3	2.2	65	632	10.3 (8.2 - 12.9)
	4	13	62	1.2	5.4	4.5	94	905	10.4 (8.6 - 12.5)
	5	13	62	1.1	5.0	4.5	84	915	9.2 (7.5 - 11.2)
	6	13	69	1.0	2.6	2.6	93	906	10.3 (8.5 - 12.4)
	7	13	69	1.0	2.9	2.9	118	887	13.3 (11.2 - 15.7)
	Mean (SD)								*10.0 (SD = 3.4)*
280	1	1	55	1.0	1.0	1.0	156	1406	11.1 (9.6 - 12.9)
	2	2	64	2.2	1.5	0.7	103	360	28.6 (24.2 - 33.5)
	3	2	64	1.5	2.4	1.6	209	794	26.3 (23.5 - 29.6)
	4	3	71	0.7	4.0	5.7	131	907	14.4 (12.3 - 16.9)
	5	3	71	1.0	5.0	5.0	109	787	13.9 (11.6 - 16.4)
	6	3	78	1.4	4.2	3.0	27	973	2.8 (1.9 - 4.0)
	7	3	78	0.6	2.1	3.5	49	884	5.5 (4.2 - 7.3)
	8	4	85	1.4	3.5	2.5	12	998	1.2 (0.7 - 2.1)
	9	4	85	0.6	5.3	8.8	56	943	5.9 (4.6 - 7.6)
	Mean (SD)								*12.2 (SD = 9.8)*

The table shows exact counts and 95% confidence intervals of the GCR estimates using Method 1 (with Pfs16 staining) as plotted and analysed in Figure 3 and Table 1. For each

replicate assay the number of days the isolate had been in culture after isolation from the patient is shown, as well as the parasitaemia in the day of the setup of the experiment (cycle before testing for proportions of gametocytes), the parasitaemia in the cycle during which the parasites were tested for expression of Pfs16, and the fold change in parasitaemia between these successive cycles. The assay replicates are presented in the order in which they were performed (the duration of time in culture occasionally does not exactly follow the same sequential temporal order for Isolates 292 and 293 as an intermediate cryopreservation and thawing of the isolates was performed). Erythrocytes from 15 different erythrocyte donors used at various times throughout the series of experiments shown here and in Supplementary Table S5 are numbered (RBC Batch), and inspection of the data indicates that these did not determine the variation between assay replicates.

Supplementary Table S4. Pairwise tests for significant differences in gametocyte conversion rates between different Ghanaian *P. falciparum* clinical isolates.

Clinical Isolates	272	280	289	292	293	296
272						
280	0.1447					
289	0.1797	0.0663				
292	0.5594	0.2523	0.0350*			
293	0.0649	0.7756	0.0043**	0.1375		
296	0.0221*	0.7577	0.0047**	0.0379*	0.7308	

P values are from Mann-Whitney comparisons of all of the individual biological replicate measures for each of the six isolates (as shown in Supplementary Table S3). Statistically significant comparisons are highlighted in green.

Supplementary Table S5. Multiple biological replicate assays of gametocyte conversion rates (GCR) for each of 13 long-term culture adapted *P. falciparum* lines of diverse origins.

Line	Replicate	RBC batch	% Parasitaemia cycle before	% Parasitaemia post-invasion	fold-change	Pfs16 +ve	DAPI +ve	GCR (95% CI)
F12	1	3	-	3.4	-	0	1000	0 (0)
	2	3	-	2.2	-	0	1000	0 (0)
	3	4	-	2.2	-	0	1023	0 (0)
	4	10	1.3	8.7	6.7	0	394	0 (0)
	5	11	2.7	3.0	1.1	0	600	0 (0)
	6	11	1.2	5.1	4.3	0	711	0 (0)
	7	11	1.3	2.2	1.7	0	380	0 (0)
	Mean							0
D10	1	2	0.8	1.7	2.2	0	515	0 (0)
	2	2	-	15	-	1	999	0.1 (0 - 5.6)
	3	2	-	4.5	-	10	1177	0.8 (0.5 - 1.6)
	4	3	-	11.0	-	2	997	0.2 (0.1 - 0.7)
	5	3	-	9.6	-	2	1000	0.2 (0.1 -0.7)
	6	9	3.4	8.8	2.6	3	997	0.3 (0.1 - 0.9)
	7	9	2.7	9.4	3.5	2	997	0.2 (0.1 - 0.7)
	Mean							0.26
T9/96	1	12	0.8	1.0	1.3	6	993	0.6 (0.3 - 1.3)
	2	13	1.1	5.3	4.8	2	733	0.3 (0.1 - 1.0)
	3	13	1.5	4.5	3.0	3	992	0.3 (0.1 - 0.9)
	4	13	2.2	7.6	3.5	2	703	0.3 (0.1 - 1.0)
	5	14	0.6	12.5	21	2	997	0.2 (0 - 0.7)
	6	14	0.6	7.8	13	3	433	0.7 (0.2 - 2.0)
	Mean							0.39
Palo Alto	1	6	0.8	1.0	1.3	12	987	1.2 (0.7 - 1.2)
	2	7	1.8	2.7	1.5	14	514	2.7 (1.6 - 4.5)
	3	7	1.7	2.2	1.3	5	321	1.6 (0.7 - 3.6)
	4	9	-	4.0	-	1	998	0.1 (0 - 0.6)
	5	9	1.4	2.9	2.1	3	996	0.3 (0.1 - 0.9)
	6	9	0.6	1.4	2.3	1	998	0.1 (0 - 0.6)
	Mean							1.00
3D7	1	2	-	9.3	-	20	980	2.0 (1.3 - 3.1)
	2	3	-	8.6	-	3	994	0.3 (0.1 - 0.9)
	3	3	-	8.4	-	12	979	1.2 (0.7 -2.1)
	4	4	0.8	2.6	3.3	20	977	2.0 (1.3 -3.1)
	5	4	-	2.2	-	17	916	1.9 (1.2 - 3.0)
	6	12	0.9	1.1	1.2	20	885	2.3 (1.5 - 3.5)
	7	12	1.1	1.4	1.3	16	983	1.6 (1.0 - 2.6)
	Mean							1.62

	1	5	-	3.5	-	83	920	9.0 (7.3 -11.1)
	2	5	-	2.5	-	16	983	1.6 (1.0 - 2.6)
	3	5	-	2.2	-	6	885	0.7 (0.3 - 1.5)
D6	4	9	1.7	3.5	2.1	28	559	5.0 (3.5 - 7.1)
	5	9	1.5	2.5	1.7	86	905	9.5 (7.8 - 11.6)
	6	9	1.0	0.1	0.1	26	973	2.7 (1.8 - 3.9)
	7	9	1.4	0.7	0.5	50	950	5.3 (4.0 - 6.9)
	Mean							4.70
	1	6	1.4	3.3	2.4	22	978	2.2 (1.5 - 3.4)
	2	6	0.8	1.5	1.9	85	914	9.3 (7.6 -11.4)
	3	6	2.5	1.5	0.6	92	917	10.0 (8.3 - 12.2)
GB4	4	6	1.2	1.0	0.8	37	964	3.8 (2.8 - 5.3)
	5	7	0.9	7.7	8.6	59	940	6.3 (4.9 - 8.0)
	6	7	1.4	3.6	2.6	16	983	1.6 (1.0 - 2.6)
	7	9	0.9	2.2	3.7	34	969	3.5 (2.5 - 4.9)
	Mean							5.26
	1	2	0.9	3.7	4.1	130	902	14.4 (12.3 - 16.9)
	2	3	1.5	1.9	1.6	4	995	0.4 (0.2 - 1.0)
	3	3	1.6	3.0	1.9	9	986	0.9 (0.5 - 1.7)
Dd2	4	4	-	1.1	-	46	953	4.8 (3.6 - 6.4)
	5	4	-	2.6	-	84	916	9.2 (7.5 - 11.2)
	6	12	-	3.6	-	23	532	4.3 (2.9 -6.4)
	7	12	-	2.1	-	35	885	4.0 (2.9 - 5.5)
	Mean							5.42
	1	2	-	9.2	-	50	691	7.2 (5.5 - 9.4)
HB3	2	2	-	5.0	-	103	1000	10.3 (8.6 - 12.3)
	3	9	3.4	4.5	1.3	57	942	6.1 (4.7 -7.8)
	4	9	0.9	7.5	8.3	56	944	5.9 (4.6 -7.6)
	Mean							6.87
	1	2	-	2.3	-	225	1022	22.0 (19.6 - 24.7)
	2	2	-	3.7	-	182	1013	18.0 (15.7 - 20.5)
	3	3	-	6.8	-	73	926	7.9 (6.3 - 9.8)
NF54	4	3	-	6.2	-	54	925	5.8 (4.5 - 7.5)
	5	13	-	1.1	-	41	619	6.6 (4.9 - 8.9)
	6	13	-	8.4	-	11	975	1.1 (0.6 - 2.0)
	7	13	-	8.8	-	27	975	2.8 (1.9 - 4.0)
	Mean							9.18

RO33	1	5	-	2.7	-	153	861	17.8 (15.4 - 20.5)
	2	5	-	1.1	-	68	943	7.2 (5.7 - 9.0)
	3	7	1.3	1.1	0.8	70	932	7.5 (6.0 - 9.4)
	4	7	2.4	0.7	0.3	92	910	10.1 (8.3 - 12.2)
	5	10	-	4.0	-	72	921	7.8 (6.3 - 9.7)
	6	10	1.4	3.6	2.6	124	875	14.2 (12.0 - 16.6)
	Mean							10.77
FCC2	1	5	2.1	4.3	2.0	211	785	26.9 (23.9 - 30.1)
	4	5	2.5	3.9	1.6	147	835	17.6 (15.2 - 20.3)
	5	5	1.1	4.8	4.4	16	484	3.3 (2.0 - 5.3)
	6	5	0.8	3.0	3.8	23	503	4.6 (3.1 - 6.8)
	7	10	-	4.8	-	69	935	7.4 (5.9 - 9.2)
	8	10	-	9.1	-	47	952	4.9 (3.7 - 6.5)
	Mean							10.78
7G8	1	6	2.1	3.2	2.7	159	856	18.6 (16.1 - 21.3)
	2	6	1.2	2.5	2.1	199	803	24.8 (21.9 - 27.9)
	3	10	0.7	1.5	2.1	135	452	29.9 (25.8 - 34.2)
	4	10	1.9	0.9	1.7	24	339	7.1 (4.8 - 10.3)
	5	10	2.2	2.6	1.2	9	456	2.0 (1.0 - 3.7)
	6	11	1.5	2.2	1.5	46	953	4.8 (3.6 - 6.4)
	Mean							14.52

Exact counts and 95% confidence intervals of the GCR estimates were performed using Method 1 (with Pfs16 staining) as plotted and analysed in Figure 4. The parasitaemia in the day of the setup of each assay (cycle before testing for proportions of gametocytes) is shown, along with the parasitaemia in the cycle during which the parasites were tested for expression of Pfs16, and the fold change in parasitaemia between these successive cycles, except for missing values for some replicates. Erythrocytes from fifteen different erythrocyte donors used at various times throughout the series of experiments shown here and in Supplementary Table S3 are numbered (RBC Batch), and inspection of the data shows that these did not determine the variation between assay replicates.

Supplementary Table S6. Pairwise tests for significant differences in gametocyte conversion rates between different *P. falciparum* laboratory lines. P values are from Mann-Whitney comparisons of all of the individual biological replicate measures for each of the 13 lines (as shown in Supplementary Table S5). Statistically significant comparisons are highlighted in green.

Lab Isolates	F12	D10	T9/96	Palo Alto	3D7	D6	GB4	Dd2	HB3	NF54	RO33	7G8	FCC2
F12													
D10	0.0047**												
T9/96	0.0022**	0.1696											
Palo Alto	0.0022**	0.2459	0.6082										
3D7	0.0012**	0.0017**	0.0052**	0.1521									
D6	0.0012**	0.0012**	0.0023**	0.0216*	0.0781								
GB4	0.0012**	0.0006***	0.0012**	0.0047**	0.0117*	0.6457							
Dd2	0.0012**	0.0012**	0.0047**	0.0338*	0.1282	>0.9999	>0.9999						
HB3	0.0048**	0.0030**	0.0095**	0.0061**	0.0012**	0.2303	0.4121	0.3124					
NF54	0.0012**	0.0006***	0.0012**	0.0082**	0.0175*	0.3176	0.535	0.3176	0.9273				
RO33	0.0022**	0.0012**	0.0022**	0.0022**	0.0012**	0.035*	0.035*	0.0734	0.026*	0.4452			
7G8	0.0022**	0.0012**	0.0022**	0.0043**	0.0082**	0.1807	0.1807	0.1101	0.3939	0.4452	>0.9999		
FCC2	0.0022**	0.0012**	0.0022**	0.0022**	0.0012**	0.336	0.2949	0.2343	0.9143	0.9452	0.3939	0.6991	

Supplementary Table S7. Numerical counts and calculated values of assays measuring the effect of choline-free medium on gametocyte conversion rates (GCR) of five different *P. falciparum* lines. The effect on GCR within each biological replicate assay is shown as a Rate Ratio with 95% confidence intervals, based on the GCR (proportions of parasites expressing Pfs16) with and without choline. Mantel-Haenszel adjusted mean Rate Ratios show the overall effects across all replicates with 95% confidence intervals.

Replicate Assays	Condition	% Parasitaemia cycle before	% Parasitaemia Post invasion	Fold-change	Pfs16 +ve	Pfs16 -ve	Rate Ratio (± 95%CI)	GCR (± 95%CI)	Adjusted mean Rate Ratio (± 95%CI)
***P. falciparum* line HB3**									
R1	-choline	2.8	3.4	1.2	16	967	2.03 (0.93 - 5.46)	1.6 (1.0 - 2.6)	**1.98 (1.55 - 2.52)**
	+ choline		3.5	1.3	7	864		0.8 (0.4 - 1.6)	
R2	-choline	0.8	9.7	12	29	943	3.69 (1.69 - 8.04)	3.0 (2.1 - 4.3)	
	+ choline		8.8	11	8	983		0.8 (0.4 - 1.6)	
R3	-choline	0.9	-	-	34	902	12.05 (3.71 - 39.13)	3.6 (2.6 -5.0)	
	+ choline		-	-	3	993		0.3 (0.1 - 0.9)	
R4	-choline	1.4	7.4	5.3	36	929	2.01 (1.15 - 3.51)	3.7 (2.7 - 5.1)	
	+ choline		4.1	2.9	18	950		1.9 (1.2 - 2.9)	
R5	-choline	0.6	5.4	9.0	24	954	1.26 (0.70 - 2.30)	2.5 (1.7 - 3.6)	
	+ choline		9.0	15	19	961		1.9 (1.2 - 3.0)	
R6	-choline	0.6	8.6	14	14	970	1.00 (0.48 - 2.09)	1.4 (0.8 - 2.4)	
	+ choline		10.0	17	14	971		1.4 (0.8 - 2.4)	
R7	-choline	0.6	2.8	4.7	20	959	0.95 (0.52 - 1.74)	2.0 (1.3 - 3.1)	
	+ choline		1.9	3.2	21	957		2.1 (1.4 - 3.3)	
R8	-choline	-	4.7	-	19	962	2.40 1.06 - 5.45)	1.9 (1.2-3.0)	
	+ choline		5.2	-	8	983		0.8 (0.4 - 1.6)	
***P. falciparum* line Dd2**									
R1	-choline	1.0	2.5	2.5	194	619	5.58 (4.03 - 7.71)	23.9 (21.1 - 26.9)	**3.51 (3.00 - 4.11)**
	+ choline		3.2	3.2	41	917		4.3 (3.2 - 5.8)	
R2	-choline	0.8	5.1	6.4	42	1867	0.95 (0.61 - 1.47)	2.2 (1.6 - 3.0)	
	+ choline		1.9	2.4	37	1562		2.31 (1.7 - 3.2)	
R3	-choline	2.1	3.8	1.8	51	912	1.21 (0.81 - 1.80)	5.3 (4.1 - 6.9)	
	+ choline		2.3	1.1	42	915		4.4 (3.3- 5.9)	
R4	-choline	1.3	7.8	6.0	105	799	16.56 (7.75 - 35.40)	11.6 (9.7 - 13.9)	
	+ choline		7.9	6.1	7	991		0.7 (0.3 - 1.4)	
R5	-choline	1.5	6.4	4.3	141	847	12.82 (6.98 - 23.53)	14.3 (12.2 - 16.6)	
	+ choline		12.5	8.3	11	977		1.1 (0.6 - 2.0)	
R6	-choline	-	9.7	-	93	824	2.07 (1.47 - 2.90)	10.1 (8.4 - 12.3)	
	+ choline		9.5	-	47	910		4.9 (3.7 - 6.5)	

P. falciparum **line 3D7**									
R1	-choline	2.8	10	3.6	17	965	1.22 (0.60 - 2.45)	1.7(1.1 - 2.8)	**1.50 (1.16 - 1.94)**
	+ choline		2.6	0.9	14	969		1.4 (0.9 - 2.4)	
R2	-choline	0.8	8.5	11	1	997	0.20 (0.02- 1.70)	0.1 (0.0 - 0.6)	
	+ choline		8.6	11	5	989		0.5 (0.2 - 1.2)	
R3	-choline	0.9	-	-	6	987	3.01 (0.61 - 14.88)	0.6 (0.3 -1.3)	
	+ choline		-	-	2	995		0.2 (0.0 - 0.7)	
R4	-choline	1.4	-	-	34	931	3.45 (1.72 - 6.95)	3.5 (2.5 - 4.9)	
	+ choline		-	-	10	970		1.0 (0.6 - 1.9)	
R5	-choline	0.6	4.5	7.5	4	940	0.82 (0.22 - 3.04)	0.4 (0.2 - 1.1)	
	+ choline		4.4	7.3	5	961		0.5 (0.2 - 1.2)	
R6	-choline	0.6	9.5	16	6	987	0.65 (0.23 - 1.82)	0.6 (0.3 - 1.3)	
	+ choline		8.8	15	9	961		0.9 (0.5 - 1.8)	
R7	-choline	0.6	-	-	35	931	1.86 (1.08 - 3.24)	3.6 (2.6 - 5.0)	
	+ choline		-	-	19	961		1.9 (1.2 -3.0)	
R8	-choline	-	5.3	-	43	913	1.28 (0.83 - 1.99)	4.5 (3.4 - 6.0)	
	+ choline		4.0	-	34	938		3.5 (2.5 - 4.8)	
P. falciparum **line T9/96**									
R1	-choline	2.2	9.6	4.4	7	985	1.00 (0.35 - 2.84)	0.7 (0.3 - 1.4)	**0.8 (0.51 - 1.19)**
	+ choline		2.2	1.0	7	985		0.7 (0.3 - 1.4)	
R2	-choline	1.5	8.4	5.6	2	995	0.57 (0.10 - 3.10)	0.2 (0.0 - 0.7)	
	+ choline		8.6	5.7	4	1131		0.4 (0.1 - 0.9)	
R3	-choline	2.2	8.1	3.7	2	995	no estimate	0.2 (0.0 - 0.7)	
	+ choline		7.2	3.3	0	999		0 (0)	
R4	-choline	0.6	-	-	2	934	0.26 (0.06 - 1.24)	0.2 (0.0 - 0.8)	
	+ choline		-	-	8	983		0.8 (0.4 - 1.6)	
R5	-choline	0.6	-	-	5	643	0.83 (0.23 - 3.09)	0.8 (0.3 - 1.8)	
	+ choline		-	-	4	429		0.9 (0.4 - 2.4)	
R6	-choline	-	4.3	-	11	978	0.84 (0.38 - 1.88)	1.1 (0.6 - 2.0)	
	+ choline		4.8	-	13	973		1.3 (0.8 - 2.2)	
R7	-choline	-	2.7	-	9	739	0.79 (0.34 - 1.87)	1.2 (0.6 - 2.3)	
	+ choline		1.6		12	780		1.5 (0.9 -2.6)	

***P.falciparum* line NF54**									
R1	-choline	-	3.7	-	27	945	2.50 (1.25 - 5.01)	2.8 (1.9 - 4.0)	**2.41 (1.97 - 2.96)**
	+ choline		12.7	-	11	978		1.1 (0.6 - 2.0)	
R2	-choline	-	6.6	-	30	943	5.11 (2.13 - 12.24)	3.1 (2.2- 4.4)	
	+ choline		6.8	-	6	990		0.6 (0.3 - 1.3)	
R3	-choline	1.5	5.1	3.4	40	819	3.53 (1.90 - 6.56)	4.7 (3.4- 6.3)	
	+ choline		4.0	2.7	13	973		1.3 (0.8 - 2.2)	
R4	-choline	1.0	4.5	4.6	43	913	5.57 (2.63 - 11.79)	4.5 (3.4 - 6.0)	
	+ choline		10.6	11	8	983		0.8 (0.4 - 1.6)	
R5	-choline	0.4	4.3	11	70	861	1.52 (1.06 - 2.18)	7.5 (6.0 - 9.4)	
	+ choline		5.0	13	47	905		4.9 (3.7 - 6.5)	
R6	-choline	0.7	5.6	8.0	60	880	1.86 (1.23 - 2.83)	6.4 (5.0 - 8.1)	
	+ choline		2.1	3.0	33	933		3.4 (2.4 - 4.8)	
R7	-choline	1.0	3.9	3.9	8	983	1.60 (0.53 - 4.87)	0.8 (0.4 - 1.6)	
	+ choline		1.6	1.6	5	986		0.5 (0.2 - 1.2)	
R8	-choline	0.6	2.5	4.2	15	970	5.06 (1.47 - 17.41)	1.5 (0.9 - 2.5)	
	+ choline		4.0	6.7	3	993		0.3 (0.1 - 0.9)	
